# Incorporating Environmental Health in Clinical Medicine

**DOI:** 10.1155/2012/103041

**Published:** 2012-05-17

**Authors:** Stephen J. Genuis, Margaret Sears, Gerry Schwalfenberg, Janette Hope, Robin Bernhoft

**Affiliations:** ^1^Faculty of Medicine & Dentistry, University of Alberta, Edmonton, AB, Canada T6G 2R7; ^2^Children's Hospital of Eastern Ontario Research Institute, Ottawa, ON, Canada K1H 8L1; ^3^American Academy of Environmental Medicine, Wichita, KS 67206, USA

What are the objectives of 21st century healthcare? Five fundamental pillars and presuppositions appear to underscore the provision of clinical healthcare, whether conventional or alternative:

- Healthcare should genuinely assist individuals
  - to prevent illness through health education;
  - to overcome and recover from disease and suffering when possible;
  - to receive compassionate and ongoing care when cure is no longer a reality.
- Healthcare should be based on credible scientific evidence rather than ideology, anecdote, or corporate science.
- Healthcare professionals should be competent, compassionate, and accountable for the care they provide.
- Outcome measures of healthcare approaches should be in place to determine evidence-based success and to protect the public interest.
- Ongoing research to study proposed interventions is in the public interest and is required for the advancement of clinical care.

While providers of clinical care, whatever the stripe, may share the same vision and objectives, twenty-first century healthcare is increasingly fragmented. We now have medicine with myriad descriptors: conventional, alternative, naturopathic, holistic, ayurvedic, traditional Chinese, osteopathic, integrative, restorative, functional, homeopathic, and so on. The potpourri of healthcare disciplines and labels can be confusing and it is challenging to recount the ideology and practices peculiar to each faction. Each discipline brings something unique to the table and proponents often advance their particular interpretation of science and their interventions as the best way to deliver health and healing to the suffering masses.

Yet, despite noble intentions and sincere aspirations by clinical practitioners from the differing groups, there is also escalating derision between assorted disciplines. Conventional and nonallopathic factions often accuse each other of lacking scientific credibility, of being deficient in evidence for various interventions, and of failing the public interest. All the while, rates of chronic mental and physical illness in both adults and children continue to escalate and to incapacitate so many suffering people [1, 2].

So with the ongoing dispersion of medical approaches, the rancour between competing ideologies, and the challenging state of health in much of the world's population, why do we need yet another branch of clinical medicine and why does the Journal of Environmental and Public Health publish a special issue to introduce and advance the clinical field of “Environmental Medicine”? We endeavour to answer such questions in this publication.

At the outset of the issue, S. Genuis aims to provide historical and contemporary evidence for the clinical field of environmental medicine as the preferred scientific approach to healthcare in an introductory piece entitled “What's out there making us sick?” An eclectic collection of papers follow that explore varied aspects of this emerging discipline and that attempt to bridge the gap between established research in relation to environmental health science and determinants of disease, and the day-to-day patient encounters in clinical practice. In selecting articles, we have endeavoured to be relevant and contemporary by remaining attuned to modern trends as well as providing a scholarly forum for the expression of novel and controversial developments, presented to standards of peer-reviewed scientific publication. We believe that presenting ideas and proposed clinical strategies for scrutiny and debate is healthy.

For example, we have a clinically relevant research article by D. Kennedy et al. reporting on the scientific efficacy of a widely popular detoxification strategy called “Ionic footbaths,” we present a manuscript exploring the clinical usefulness of sweating as a means to eliminate accrued toxicants, as well as providing a practical paper by D. Colson on the safe removal of dental amalgam. Furthermore, we have accepted a provocative review piece by a number of scientists on research relating to a phenomenon they call “Earthing” as well as a paper that introduces an unconventional yet apparently successful clinical approach to assisting patients with bone compromise. We are not promoting or endorsing any specific clinical therapy or intervention. Rather, we believe that scientific censorship is dangerous and that the broad spectrum of therapies should be critically assessed equally and evaluated based on scientific merit, not on the medical paradigm “box” to which they are ascribed.

*From the Lead Editor*

Each of the guest editors kindly responded to an invitation to submit an article for independent peer review. Dr. Margaret Sears presents an article discussing practical aspects of the environmental health field while a piece by Dr. Gerry Schwalfenberg highlights the profound clinical worth of vitamin D in healthcare. A manuscript by Dr. Robin Bernhoft gives an overview of the challenges and clinical management of mercury exposure. A couple of interesting papers including a piece by Dr. Janette Hope et al. draw attention to the serious and widespread clinical problem of mold and mycotoxins among exposed individuals and groups.

The publishers at the Journal of Environmental and Public Health deserve much credit for recognizing the importance of this expanding field and for inviting a special issue exploring the discipline of clinical environmental medicine. It is by initiatives such as this that the translation of new knowledge occurs and that innovative clinical trends are established. Challenging the status quo with the adoption of new ideas and skills has always been and remains the path to scientific and clinical progress.

As lead editor of this issue, I must admit there was reservation when we first embarked on this expedition. While there are many scientific researchers in the burgeoning field of environmental science and an ever-expanding number of scientific journals that focus on this subject, there is a paucity of clinicians worldwide who have both the knowledge and skills to assimilate information from environmental health research and translate it into clinical practice, and even fewer with the ability and commitment to prepare scientific articles. With that realization, I was concerned we might receive minimal response to the call for papers. I was wrong: the overwhelming response and submission of articles for this issue has been heart warming and exemplifies the mounting interest in this field. The time and work involved has been well worth it and I am most grateful to my fellow guest editors, who have been extraordinarily helpful in the process.

In the end, however, I remain disenchanted by the mounting divisions within the health care field. I am not fond of labels and disunity; I prefer medicine without descriptors. Scientific clinical medicine should be based on credible untainted research and reporting, reproducible observation, as well as competent and compassionate health care in order to provide favorable outcomes for patients and populations. I contend that it is time to incorporate credible research science and emerging evidence, whatever the source, into the practice of mainstream clinical medicine. It is to this end that this special issue has come forward. Thank you for your interest in this publication we have prepared.
